# Gi-protein-coupled β _1_-adrenergic receptor: re-understanding the selectivity of β _1_-adrenergic receptor to G protein: Gi-protein-coupled β _1_-adrenergic receptor

**DOI:** 10.3724/abbs.2022096

**Published:** 2022-08-08

**Authors:** Hao Chen, Suli Zhang, Ruiqi Hou, Huirong Liu

**Affiliations:** 1 Department of Physiology & Pathophysiology School of Basic Medical Sciences Capital Medical University Beijing 100069 China; 2 Beijing Key Laboratory of Metabolic Disorders Related Cardiovascular Disease Capital Medical University Beijing 100069 China

**Keywords:** β _1_-adrenergic receptor, Gi protein, G protein switch, signal transduction, cardioprotective effect

## Abstract

β _1_-adrenergic receptor (β _1_-AR), a member in the family of G-protein-coupled receptors, is a transmembrane receptor of great significance in the heart. Physiologically, catecholamines activate β _1_-AR to initiate a positive chronotropic, inotropic, and dromotropic change. It is believed that β _1_-AR couples to Gs protein and transmits the signal through second messenger cAMP. However, increasing research shows that β _1_-AR can also bind with Gi protein in addition to Gs. When β _1_-AR-Gi is biasedly activated, cardioprotective effects are introduced by the activated cGMP-protein kinase G (PKG) pathway and the transactivation of epidermal growth factor receptor (EGFR) pathway. The discovery of β _1_-AR-Gi signaling makes us reconsider the selectivity of G protein with regard to β _1_-AR, which also provides new ideas for the treatment of heart diseases. This review summarizes the discovery of β _1_-AR-Gi pathway, including the evidence that supports β _1_-AR’s capability to couple Gi, details of the transduction process and functions of the β _1_-AR-Gi signaling pathway.

## Introduction

G-protein-coupled receptors (GPCRs) are the largest family of membrane proteins in the human body, which transmit extracellular information into the intracellular signaling [1]. β _1_-adrenergic receptor (β _1_-AR) is a kind of GPCRs widely distributed in the cardiovascular system. In the heart, β _1_-AR accounts for approximately 80% of β-adrenergic receptors, with the remaining 20% belonging to β _2_ adrenergic receptor (β _2_-AR) and β _3_ adrenergic receptor (β _3_-AR) respectively [ 2, 3] . Under physiological conditions, β _1_-AR is a critical receptor protein regulating cardiac function. β _1_-AR can be activated by catecholamines, such as norepinephrine (NE) and epinephrine, to play the role of positive inotropic, positive chronotropic and positive dromotropic action to meet the needs of the body’s fight-or-flight response [3]. In pathological states, β _1_-AR is also the target of drug therapy for heart diseases [4]. Beta blockers, the inhibitor of β _1_-AR such as propranolol, metoprolol and carvedilol, can be used for the treatment of heart failure, coronary heart disease, atrial fibrillation, *etc*. [ 5– 7] .

G protein, a guanine-nucleotide-binding protein, is a trimeric complex comprising subunits of Gα, Gβ, and Gγ. According to the variations of the α subunits, G protein can be divided into Gs, Gi, Gq/11, and G12/13 [ 8– 10] . Traditionally, β _1_-AR is regarded to couple only Gs [ 11– 13] . Activated β _1_-AR binds to the Gs protein and induces the synthesis of the second messenger cyclic adenosine monophosphate (cAMP) catalyzed by adenylate cyclase (AC), which phosphorylates downstream proteins through protein kinase A (PKA) to complete signal transduction [14]. Meanwhile, after Gs binds to activated receptor, Gβγ subunit dissociates from Gαs subunit and G protein coupled receptor kinase (GRK) is recruited to catalyze receptor phosphorylation [ 8, 15] . GTP binding induces the separation of Gs from the receptor [8].

Yet, increasing studies have shown that resembling β _2_-AR, β _1_-AR can also couple Gi protein in addition to Gs [ 12, 16] . Recent research has shown that carvedilol biasedly activates the β _1_-AR-Gi pathway and exerts cardioprotective effects through cyclic guanosine monophosphate (cGMP)-protein kinase G (PKG) pathway [17]. Moreover, structural analysis of the β _1_-AR-Gi complex also underlies a solid foundation for research of the complex [10].

Here, we use the beginning and end of β _1_-AR-Gi signaling as a clue, reviewing the course of the β _1_-AR-Gi pathway discovery from the very evidence of β _1_-AR coupling Gi to the process and function of β _1_-AR-Gi signal transduction, while looking forward to the future β _1_-AR-Gi research directions.

## Evidence for Gi Coupling β _1_-AR

### Early exploration of Gi coupled β _1_-AR

In 2002, researchers found that β _1_-AR has the ability to couple Gi by using neonatal myocytes from β _1_-AR knockout (KO), β _2_-AR KO, and β _1/2_-AR KO mice [12]. However, this feature is usually not manifested due to the existence of PDZ domain binding motif at the C-terminal of β _1_-AR and PDZ domain-containing scaffolding proteins, through which the coupling of β _1_-AR and Gi is limited. PDZ domain binding motif mediates specific protein reactions and is, in essence, a protein module with a conserved structure [18]. In this study, the researchers mutated the mouse β _1_-AR PDZ domain binding motif ~ESKV~ into ~EAAA~ to form the mutant β _1_-AR (β _1_-AR-PDZ). Subsequently, HA-labeled mouse wild-type β _1_-AR or β _1_-AR-PDZ was transfected into β _1/2_-AR KO mouse neonatal myocytes, and their contraction rate was recorded. The results demonstrated that the contraction rate of isoproterenol (ISO) was first increased and then decreased below the basic level, showing a biphasic contraction rate response, which was consistent with the results shown in β _1_-AR KO mouse neonatal myocytes expressing β _2_-AR. All of above phenomenon can be blocked by Gi inhibitor pertussis toxin (PTX).

β _2_-AR is another β-adrenergic receptor in cardiomyocytes that couples Gs and Gi [ 19, 20] . Additionally, β _2_-AR KO myocytes were treated with a membrane permeable polypeptide Tag-β _1_PDZ (466GRQGFSSESKVCOO~ of β _1_-AR) that mimics the binding mode of β _1_-AR PDZ domain, and ISO stimulation also caused a biphasic contraction response. This effect was inhibited by PTX as well [12]. In general, the above series of results suggest that β _1_-AR has the potential to couple the Gi protein, but the presence of PDZ restricts the binding of β _1_-AR to Gi.

In 2004, Lefkowitz’s group [21] showed that the physiological ligand of β _1_-AR, NE, can also active the β _1_-AR-Gi pathway. It was found that in Chinese hamster ovary (CHO) cells, when overexpressed β _1_-AR was stimulated by NE, ERK activity was increased significantly, which could be completely blocked by PTX.

### The clarity of direct evidence

In 2017, researchers directly validated Gαi binding to β _1_-AR when stimulated by carvedilol using the proximity ligation assay (PLA) and co-immunoprecipitation (CO-IP) [16]. Carvedilol is a β-blocker that can biasedly activate β _1_-AR-β-arrestin signaling [22]. In HEK293 cells overexpressing the Flag-β _1_-AR, with the increase of carvedilol concentration (10 ^–9^–10 ^–5^ M), the amount of Gi coupling β _1_-AR gradually increased in a concentration-dependent manner. However, ISO did not trigger β _1_-AR to Gi binding even in the concentration range of 10 ^–9^ M to 10 ^–5^ M. Forby, the β _1_-AR-Gi interaction was detected after treating HEK293 cells with 10 ^–5^ M carvedilol for 5 min. This is, in fact, time-dependent, as more Gi is bound to β _1_-AR in a prolonged stimulation time (30 min) [16]. The binding of β _1_-AR to Gi, detected by either PLA or CO-IP experiments, could be blocked by PTX [16], which confirmed that carvedilol is a β _1_-AR-Gi-biased ligand and promotes the binding in concentration-dependent and time-dependent manners.

### Analysis of the structure and function of β _1_-AR-Gi

In 2021, Xiang’s group [17] found that carvedilol repaired the heart function damage in mice fed with high-fat diet, and, correspondingly, that the protective effect of carvedilol was reversed by PTX. Apart from further evidence that Gi couples β _1_-AR, this study also confirmed the cardioprotective effect of β _1_-AR-Gi activation in animal models of cardiac dysfunction. Almost simultaneously, Huang’s group [10] analyzed the structure of the ISO-β _1_-AR-Gi complex by cryo-electron microscopy with a resolution of 3.0 Å. Together, these results suggest that Gi can couple β _1_-AR in response to agonist or blocker stimulation.

But still, some confusions need to be clarified in order to put us in the picture. (1) How much influence does PDZ domain binding motif have on β _1_-AR-Gi binding? The PDZ domain binding motif is one of the reasons, but not the only one that is preventing the binding of β _1_-AR to Gi. This is because the PDZ is located at the C-terminal of the receptor. It has been demonstrated that the C-terminal alone is not sufficient to determine the binding of β _1_-AR to Gi [16]. Results from earlier research validated that it is the presence of β _1_-AR C-terminal PDZ domain binding motif that prevents ISO to activate β _1_-AR-Gi combination [ 12, 16] . Subsequent studies demonstrated that β _1_-AR can still bind to Gi even in the absence of external intervention on β _1_-AR PDZ domain binding motif stimulated by carvedilol [ 16, 17] . Although the ligand used in the protein purification of β _1_-AR-Gi complex is ISO, there are no PDZ domain-containing scaffolding proteins under the experimental conditions [10]. It suggests that different drugs may vary in modes that cause β _1_-AR-Gi binding. Apparently, while carvedilol causes β _1_-AR-Gi coupling after 5 min of stimulation [16], an ISO stimulation only exhibits obvious β _1_-AR-Gi binding in 15 min [12]. This phenomenon still requires in-depth research. (2) Would Gi couple β _1_-AR under physiological conditions? We speculate that NE can also induce β _1_-AR binding to Gi under physiological conditions, because it has been shown that NE can induce β _1_-AR binding to Gi in CHO cells [21]. However, there must be extremely stringent conditions for this, and the exact conditions required need to be further investigated. In the above-mentioned studies, all the combinations of β _1_-AR and Gi were formed under certain experimental conditions and disease models. Yet, does β _1_-AR also bind with Gi when catecholamines activate β _1_-AR under normal physiological conditions? That is also a question which needs to be answered in the future.

## The Selection of β _1_-AR on Gi

The switching of G protein refers to the ability of certain GPCRs to bind multiple G proteins to convert from one type of G protein to another when stimulated by a ligand. β _2_-AR is a typical representative of such kind. When β _2_-AR is activated by ISO, the receptor interacts first with the Gs protein and then transmits the signal through the AC-cAMP-PKA pathway. PKA, as a protein kinase, phosphorylates β _2_-AR, which in turn promotes the switching of β _2_-AR from Gs protein to Gi [23]. G protein switching following β _2_-AR activation was demonstrated by an experimental method of neonatal rat cardiac myocyte contraction rate, which occurred approximately 15 min after ISO stimulation [ 11, 12] .

However, the mechanism of β _1_-AR-biased binding to Gi deviates from that of β _2_-AR. In HEK293 cells, although stimulation by ISO causes β _1_-AR to bind with Gi, it is not sufficient to show that β _1_-AR can bind with Gs and Gi sequentially like β _2_-AR [12]. In addition, under the action of carvedilol, the Gs/Gi switch of β _1_-AR differs from that of β _2_-AR in HEK293 cells [16]. The principal factors of β _2_-AR G protein switching, the Gs-receptor binding and the receptor phosphorylation by PKA have little effect on the G protein switching regarding β _1_-AR. Rather, the C-terminal of β _1_-AR plays a key role in determining the binding of Gi protein to β _1_-AR. Through interchanging the C-terminal of β _1_-AR and β _2_-AR, two chimeras were obtained: β _1/2_-AR (β _1_-AR chimeric β _2_-AR C-terminal) and β _2/1_-AR (β _2_-AR chimeric β _1_-AR C-terminal). Carvedilol promotes the recruitment of Gi to β _1_-AR, but not β _2/1_-AR or β _1/2_-AR [16]. Previous studies also showed that the PDZ domain at the C-terminal of β _1_-AR hinders the binding of Gi to β _1_-AR in neonatal myocytes [12]. These results suggest that while the β _1_-AR C-terminal influences the selection of Gi by the β _1_-AR, the act is not sufficient by C-terminal alone. However, the results of Lefkowitz’s group [21] showed that PKA is involved in the Gs/Gi switching of β _1_-AR in CHO cells. It was found that in CHO cells, NE activation of β _1_-AR increased ERK activity. Both the PKA inhibitor H-89 and the Gi inhibitor PTX reversed this phenomenon. The results are different between different groups concerning the question of whether PKA affects the switching of Gs/Gi in β _1_-AR. We speculate that the difference in experimental conditions between the groups may have led to the difference in conclusions. In CHO cells, NE activation of β _1_-AR increases ERK activity. Both PKA blocker H89 and Gi blocker PTX reversed this phenomenon [21]. In HEK293 cells, carvedilol biasedly induced Gαi to bind to β _1_-AR. Mutating the PKA phosphorylation site of β _1_-AR or using H-89 did not affect the binding of Gαi to β _1_-AR ( Table 1) [16]. This indicates that the effect of PKA on Gs/Gi switching of β _1_-AR is different under different experimental conditions. But one thing for sure is that G protein is also switched after β _1_-AR activation.

**Table:** **Table 1** Comparison of experimental conditions for the detection of Gs/Gi switching of β _1_-AR

Type	Cell line	Ligand	Testing index	Reversal experiments	Ref.
PKA affects Gs/Gi switching of β _1_-AR	CHO	NE	EKR phosphorylation	Both PKA inhibitor H-89 and Gi inhibitor PTX can reverse EKR phosphorylationcaused by NE.	[21]
PKA does not affect Gs/Gi switching of β _1_-AR	HEK293	Carvedilol	Binding of Gαi to β _1_-AR	Neither mutating the PKA phosphorylationsite of β _1_-AR nor using H-89 affects thebinding of Gi to β _1_-AR.	[16]

However, the above studies were carried out in cells, and the ligand appertained to β _1_-AR is relatively uniform (only by ISO or carvedilol stimulation). In animals, however, besides the exogenous administration of ISO or carvedilol, the local catecholamines in the body are also β _1_-AR agonists. The results of carvedilol-treated mice with heart dysfunction have proved that carvedilol biasedly activates β _1_-AR-Gi and exerts cardioprotective effects through the cGMP-PKG pathway [17]. It is noteworthy that catecholamines, as orthotopic ligands, activate β _1_-AR to trigger its biased binding to Gs; carvedilol, also as an orthotopic ligand [24], rather promotes the preferential binding of β _1_-AR to Gi. Therefore, it raises a question: how does the switching of β _1_-AR-G protein engage *in vivo*? It is reasonable to speculate that Gs is coupled to β _1_-AR in the body in response to stimulation with catecholamines, yet carvedilol switches β _1_-AR-bound G protein from Gs to Gi. In this process, the C-terminal of β _1_-AR is involved in the selection of G protein. However, the specific mechanism of β _1_-AR G protein switching remains to be further explored.

## The Conformation of β _1_-AR-Gi Complex

Recently, Huang’s group [10] determined the conformation of turkey β _1_-AR-Gi complex at the resolution of 3.0 Å with the full agonist isoproterenol. The structural analysis of this complex has greatly helped us to understand the binding mode of β _1_-AR to Gi.

In this complex, β _1_-AR adopts the same active-state conformation as the β _1_-AR-Gs complex [ 10, 25] . Briefly, transmembrane 6 (TM6) is rotated outward by ~14 Å, which is the utmost structural change in the cytoplasmic side of β _1_-AR, while TM7 is moved inward by ~5 Å [25]. In addition, the conserved D(E)RY motif on TM3 and the conserved NPxxY motif on TM7 both undergo conformational changes as well, in order to couple Gi in the transducer binding pocket formed by TM3, TM5, TM6 and intracellular loop 2 (ICL2). The function of transducer binding pocket is to hold the C-terminal α5-helix of the Ras-like domain on Gα subunit. Gα, containing a Ras-like GTPase domain and an α-helical domain, is one of the three subunits of G protein. These two domains regulate the release of GDP from Gα and the binding of GTP [ 8, 26] . In the β _1_-AR-Gi complex, the α-helical domain is rotated open by ~79°, and thus is displaced ~37 Å of its mass center relative to the Ras-like GTPase domain: this is the conformational change that is of the most significance in the Gαi subunit [10]. Similarly, the conformational change of the α-helical domain also exists in a β _1_-AR-Gs complex. However, in Gs, the α-helical domain has a rotation of ~96° instead, and the distance between mass centers is ~38 Å [25].

Although β _1_-AR can bind with both Gs and Gi, the selectivity of β _1_-AR to Gs is significantly higher than that of Gi under ISO stimulation [ 10, 27] . Multiple distinct regions of β _1_-AR, like ICL2 and ICL4 (TM7/TM8 linker in C terminal), contribute to the determinants of G-protein biased selection. As a result, based on the analysis of complex conformation, the overall structure of β _1_-AR-G protein complex determines the selectivity of G protein [10].

In summary, on one hand, β _1_-AR is in an activated state under ISO stimulation, exposing the transducer binding pocket to couple Gs or Gi protein; on the other hand, carvedilol, a special beta-blocker with biased agonist activity [24], can bias the β _1_-AR to bind with Gi [ 16, 17, 24, 28] . The phenomenon is a stirring of curiosity to us: what is the conformation of the carvedilol-β _1_-AR-Gi complex? This perplexity can only be explained by analyzing the structure of the carvedilol-β _1_-AR-Gi complex. Gi coupling requires the outward movement of TM6 of β _1_-AR, which is a hallmark of GPCR activation [10]. Therefore, carvedilol-bound β _1_-AR couples to Gi, suggesting that carvedilol has the ability to induce β _1_-AR to an active conformation. Unfortunately, the structure of β _1_-AR-Gi complex with carvedilol as a ligand has not yet been resolved. Recently, human β _1_-AR was analysed [29], which provided a better structural basis for the development of drugs that promote biased agonism of β _1_-AR-Gi.

## Two β _1_-AR-Gi Signaling Pathways

### β _1_-AR-Gi-EGFR-ERK pathway

**Figure 1:**
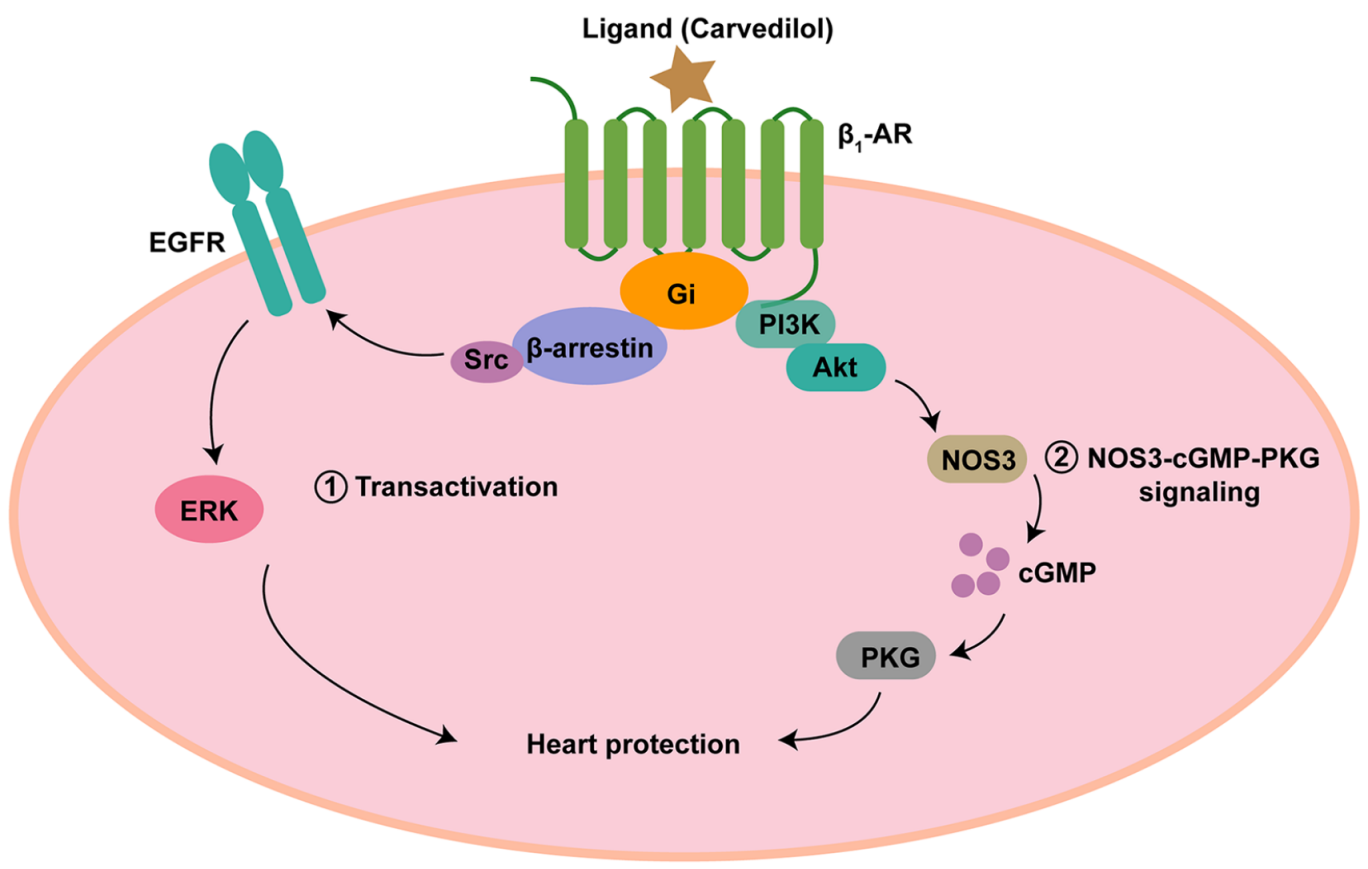
Figure 1 Different signaling pathways, same cardioprotective effects EGFR-ERK pathway and NOS3-cGMP-PKG signaling can be activated by biased activation of β 1-AR-Gi pathway. Both signaling pathways play a role in cardiac protection.

In 2008, Rockman’s group [22] provided evidence proving that carvedilol can transactivate epidermal growth factor receptor (EGFR) and its downstream signaling protein extracellular signal-regulated kinase (ERK) after biased β _1_-AR-β-arrestin activation. Ten years later, they [ 16, 22] further developed this particular signaling pathway: carvedilol activates β _1_-AR to bind Gi, and then recruits β-arrestin and Src to transactivate the EGFR-ERK pathway ( Figure 1). Additionally, β _1_-AR has been shown to activate the EGFR-ERK signaling pathway by β-arrestin transactivation in response to chronic catecholamines stress, thereby producing cardioprotective effects [ 30, 31] . These facts offer sufficient theoretical foundation for the future usage of this signal pathway to treat heart diseases.

### β _1_-AR-Gi-PI3K-Akt-NOS3-cGMP-PKG pathway

Recent research has revealed a new signaling pathway through animal and cell experiments: β _1_-AR-Gi-PI3K-Akt-NOS3-cGMP-PKG ( Figure 1) [17]. This study showed that carvedilol increased the contractile shortening in adult mice left ventricular myocytes, although this increase was only 45% of that caused by ISO. What is fascinating is that the carvedilol-induced contraction cannot be blocked by the PKA inhibitor PKI, but by the PKG blocker DT-2. Two pathways, cAMP-PKA and cGMP-PKG, are detected by fluorescence resonance energy transfer during carvedilol stimulation, showing that carvedilol significantly enhances the cGMP-PKG signal but has little effect on the cAMP-PKA signal. Moreover, the carvedilol-induced cGMP-PKG signal is blocked by Gi inhibitor PTX, PI3K inhibitor LY294002 and Akt inhibitor MK2206.

Nitric oxide synthase (NOS) is the key enzyme in nitric oxide (NO) production. NO is a promotor which triggers cGMP synthesis by activating soluble guanylyl cyclases (sGCs) [ 32, 33] . The NOSs that function in the heart are mainly NOS1 and NOS3 [34]. By interfering the expressions of NOS1 and NOS3 in adult left ventricular myocytes, the researchers revealed that carvedilol initiates cGMP signaling through the NOS3 pathway. Carvedilol, through the biased activation of the aforementioned signaling pathway, plays a cardioprotective role. Additionally, carvedilol significantly improves cardiac functions, such as increased ejection fraction, improved fraction shortening, and improved systolic shortening, in high-fat diet-induced cardiac insufficiency mice.

Although the two signal pathways are different, their function is to protect the heart. It may help clarify the mechanism of carvedilol in the treatment of heart diseases.

## Conclusions and Perspectives

As a GPCR, the key to the function of β _1_-AR lies in its binding with G protein to achieve the purpose of information transmitting [35]. In 1985, β _1_-AR was shown to bind to Gs [36]. The majority of the subsequent studies focused on the β _1_-AR-Gs pathway, for example, explaining the mechanism of β _1_-AR function [14] and verifying it as a therapeutic target for heart disease [ 37, 38] . After preliminary studies in 2002 which made it clear that β _1_-AR can actually recruit Gi, growing evidence supported that β _1_-AR can indeed bind to Gi and play a cardioprotective role through different signaling pathways [ 10, 16, 17] . The whole set of discoveries prompted us to re-evaluate the preference of β _1_-AR over G protein. Therefore, we used “G protein coupled receptor” as the word base and modified it appropriately to emphasize Gi-protein-coupled β _1_-adrenergic receptor.

Of course, there are many factors which influence the specificity selection process of G protein, such as the conformational changes of β _1_-AR [ 10, 25] , the choice of ligands (NE, ISO, and carvedilol) [ 10, 12, 17] , and PKA [ 16, 21] . Both the β _1_-AR agonists NE and ISO, and blocker carvedilol can induce β _1_-AR binding to Gi [ 10, 12, 16, 17, 21] . In addition, the β _1_-AR conformation must be suitably altered to expose the transducer binding pocket to bind with Gi proteins [10]. As for the role of PKA, the findings are contradictory in different studies. In CHO cells, PKA affects the selection of Gi by the receptor when NE activates β _1_-AR [21]. However, in HEK293 cells, the effect of PKA is trivial when carvedilol is biased to activate β _1_-AR-Gi [16]. This further suggests that the binding of β _1_-AR to Gi requires strict conditional constraints. Thus, it is a coordinated action of multiple factors that drives β _1_-AR to finally bind to Gi protein.

In the meantime, there are still many issues to be resolved in the whole process of re-evaluating the β _1_-AR-Gi coupling, including (1) the prerequisites for β _1_-AR-Gi binding; (2) the β _1_-AR-Gs/Gi switch mechanism; (3) analysis of carvedilol-β _1_-AR-Gi complex.

The discovery of the β _1_-AR-Gi pathway is exciting. The biased binding of β _1_-AR to Gi and its cardioprotective effects provide vast vistas for the treatment of cardiac conditions. Currently, the cardioprotective effect of the β _1_-AR-Gi pathway is mainly achieved by carvedilol. Clinical studies have shown that, as a beta-blocker, carvedilol is also widely used in the treatment of hypertension [ 39, 40] , cirrhosis and gastroesophageal reflux [41]. Basic studies have shown that carvedilol inhibits the progression of hepatic fibrosis in the digestive system in mice [42]. In the nervous system, carvedilol also has neuroprotective effects in diabetic neuropathy [43]. Although there is a lack of direct evidence whether carvedilol plays a part via the β _1_-AR-Gi pathway in the above diseases, the very existence of this pathway provides a theoretical basis for exploring strategies to treat these diseases. Hopefully, the treatment targeting β _1_-AR will not only block the Gs pathway, but also provide the full value of β _1_-AR-Gi pathway in bringing welfare to patients with heart diseases.
